# Neurobehavioral Comorbidities in Canine Idiopathic Epilepsy: New Insights into Cognitive and Emotional Domains

**DOI:** 10.3390/ani15111592

**Published:** 2025-05-29

**Authors:** Rosado Belén, Palacio Jorge, Menchaca Carolina, García-Belenguer Sylvia

**Affiliations:** 1 Hospital Veterinario Universidad de Zaragoza, Departamento de Patología Animal, Universidad de Zaragoza, Miguel Servet, 177, 50013 Zaragoza, Spain; jpalacio@unizar.es (P.J.); sgarcia@unizar.es (G.-B.S.); 2 Unidad Académica de Genética y Mejora Animal, Facultad de Veterinaria, Universidad de la República (UdelaR), Ruta 8 km 18, Montevideo 13000, Uruguay; cmenchaca21@gmail.com

**Keywords:** canine epilepsy, idiopathic epilepsy, drug-resistant epilepsy, neurobehavioral comorbidities, cognitive dysfunction, quality of life, cluster analysis

## Abstract

Neurobehavioral comorbidities in dogs with idiopathic epilepsy (IE) are increasingly recognized, yet their phenotypic variability and clinical implications remain poorly understood. This study aimed to identify behavioral changes following seizure onset and to explore the feasibility of stratifying patients based on neurobehavioral profiles. Seventy client-owned dogs with IE were enrolled and grouped according to treatment: 29 had drug-resistant epilepsy (DRE), 29 were drug-sensitive (DSE), and 12 remained untreated. Owners completed a modified version of the C-BARQ questionnaire, assessing behaviors before and after seizure onset. Nearly one-third of behavioral items showed significant changes, particularly in attachment and attention-seeking behaviors, separation-related behaviors, eating behavior, and signs of cognitive decline such as reduced trainability and dementia-like signs. Principal component analysis followed by cluster analysis revealed two distinct neurobehavioral profiles: Cognitive and Emotional. The Cognitive cluster was associated with a higher total questionnaire score, poorer seizure control (predominantly DRE), and lower owner-perceived quality of life. In contrast, the Emotional cluster was more frequently observed in dogs with DSE or no treatment and was associated with higher quality of life scores. These findings support the clinical relevance of behavioral stratification in canine epilepsy and underscore the need for individualized, multimodal approaches to improve patient care.

## Simple Summary

Epilepsy is one of the most common neurological conditions in dogs. In addition to seizures, many affected dogs show changes in their behavior during the periods between seizures, such as decreased trainability, increased anxiety/fear, or being unusually clingy with their owners. In this study, we examined the behavior of 70 dogs with idiopathic epilepsy, based on information provided by their owners before and after the onset of seizures. We found that some behaviors—such as difficulties in learning new tricks, separation-related problems and increased contact-seeking, changes in eating habits, and demented behaviors—became more frequent after seizures started. We then grouped the dogs based on their behavior patterns and identified two distinct profiles: one characterized by signs related to cognitive decline and another with more emotional behaviors like anxiety/fear, aggression, or attachment. These profiles were linked to how well the dogs responded to treatment and to the quality of life reported by their owners. This research shows that not all dogs with epilepsy behave the same way, and recognizing these differences can help veterinarians offer more individualized care, ultimately improving quality of life for both dogs and their families.

## 1. Introduction

Neurobehavioral comorbidities of epilepsy have been described in humans [1,2] and rodent models [3] and, more recently, in dogs [4]. The term “comorbidity” describes the co-occurrence of two distinct diseases in the same individual at a rate exceeding what would be expected by chance, thereby reflecting the clinical complexity of each patient [5]. The etiology of these comorbidities is likely multifactorial [1,6], and it remains unclear whether they represent a risk factor for the development of epilepsy, a component of the epilepsy phenotype, an adverse effect of certain antiseizure medications (ASMs), or a combination of these factors [7].

Neurobehavioral comorbidities are common in both childhood and adult epilepsy, with patients exhibiting an increased risk of developing psychiatric disorders [1]. Anxiety and depression are the most frequently reported psychiatric comorbidities, with a prevalence of 25.6% and 35%, respectively [8]. Similar prevalence rates for anxiety (15–36%) and depression (8–35%) have been observed in childhood epilepsy [9], alongside attention deficit hyperactivity disorders (ADHD, ~40%) and autism spectrum disorders (~20%) [3]. These psychiatric disorders have been linked to both disease-related and psychosocial factors [6], and there is a growing recognition of the bidirectional relationship between epilepsy and its comorbidities [3]. Furthermore, epilepsy is associated with a range of cognitive impairments, varying from mild deficits to severe dysfunction across multiple cognitive domains. Several studies suggest that these impairments are present at epilepsy onset—even before the initiation of ASM therapy—implying that the process of epileptogenesis itself may underlie cognitive compromise [1,10,11].

In canine idiopathic epilepsy (IE), several studies have reported behavioral changes following seizure onset that appear to be driven primarily by the activation of the fear/anxiety emotional system [12,13,14]. Affected dogs may also display ADHD-like signs, including increased excitability, impulsivity, chasing behavior, and inattention [14,15,16]. Additionally, cognitive impairments in these animals have been documented as reduced trainability [14,17] and spatial memory deficits [18]. Notably, the age of onset, clinical presentation, and progression of cognitive impairment in naturally occurring canine IE differ from the patterns typically observed in age-related cognitive dysfunction [19]. Despite the expanding evidence on neurobehavioral comorbidities of canine IE, significant overlap among clinical signs persists [4], underscoring the need for further research.

Emerging evidence also suggests that the response to ASMs may be linked to neurobehavioral comorbidities in dogs. For example, dogs with drug-refractory epilepsy (DRE) have been reported to show more significant increases in aggression, abnormal perception, and demented or repetitive behaviors [12], as well as lower trainability scores compared to drug-responsive counterparts [14]. Both the overall medication status and the specific ASM administered appear to influence these outcomes. One study observed improvements in fear responses and trainability following phenobarbital treatment [7], whereas another reported increased anxiety, depression, and attention-seeking behavior in dogs treated with levetiracetam [20].

Beyond the previously mentioned neurobehavioral comorbidities, the relationship between epilepsy and eating behavior has been scarcely investigated in humans [21] and remains unexplored in dogs. In humans, obesity has been described as a risk factor for epilepsy [22]. Additionally, certain ASMs—such as carbamazepine or valproate—have been used in the treatment of bulimia or anorexia nervosa in patients with epilepsy and psychiatric disorders [23]. In dogs, polyphagia is a common adverse effect of first-line ASMs, including phenobarbital, imepitoin, and potassium bromide (KBr), whereas anorexia or loss of appetite has been reported with levetiracetam and zonisamide [24]. However, aside from the medication-related side effects, it remains unknown to what extent epilepsy as a disease may affect eating behavior in dogs. Previous research has shown that dogs with medical or behavioral issues are more likely to be perceived by their owners as exhibiting emotional eating—defined as changes in food intake in response to stress or negative emotional states [25]. Therefore, further investigation into the eating behavior of dogs with IE, particularly regarding emotional eating, is warranted to elucidate possible associations.

In a recent survey, 83.6% of owners of dogs with IE reported observing interictal anxiety; however, only 28.5% felt adequately supported by their veterinarian in managing this issue [26]. Recognizing cognitive and emotional changes during interictal periods—including alterations in eating behavior—and potentially stratifying patients based on these comorbidities may facilitate the implementation of targeted behavioral therapies. Such interventions could improve quality of life (QoL), even if they do not directly impact seizure control. The present study aimed to further identify neurobehavioral comorbidities in canine IE and to explore the feasibility of classifying patients according to behavioral and clinical variables. To this end, an owner-directed questionnaire was administered to assess behavioral changes in dogs before and after the onset of seizures.

## 2. Materials and Methods

### 2.1. Ethics Statement

Before enrollment, owners received comprehensive information about the study’s objectives and procedures. They were given the opportunity to ask questions and either consent to or decline participation (see informed consent in Supplementary Materials). All procedures were conducted under Project License PI67/21, approved by the Ethics Committee for Animal Experiments at the University of Zaragoza.

### 2.2. Animals and Procedures

Seventy dogs with IE were recruited from the neurology service at the Hospital Veterinario of the Universidad de Zaragoza (Spain). The owners were invited to complete an online questionnaire to assess the prevalence and severity of various behavioral problems. Dogs were included if they met at least the Tier I confidence level criteria for an IE diagnosis. These criteria comprise a history of two or more unprovoked epileptic seizures occurring at least 24 h apart, seizure onset between six months and six years of age, unremarkable interictal physical and neurological examinations, and no abnormalities on complete blood tests and urinalysis. In instances where seizure onset occurred outside the typical age range or when polytherapy was necessary due to insufficient seizure control with a single antiepileptic medication, Tier II confidence level criteria—including a normal MRI and cerebrospinal fluid analysis—were additionally required [27]. Moreover, a minimum interval of six months between seizure onset and study enrollment was mandated for all cases.

The questionnaire was adapted from a shorter, translated Spanish version of the validated C-BARQ [28]. It comprised of 40 items distributed across eight sections, covering a total of 11 behavioral subscales: (1) learning and trainability (4 items); (2) aggression [subsections: (2a) toward family members, 4 items; (2b) toward strangers, 4 items; (2c) toward other dogs, 2 items)]; (3) fear and anxiety [subsections: (3a) to social stimuli, 4 items; (3b) fear/anxiety to non-social stimuli, 4 items)]; (4) separation-related behaviors (4 items); (5) excitability (4 items); (6) attachment and attention-seeking behavior (3 items); (7) eating behavior (5 items); and (8) repetitive or demented behaviors (2 items). The “Eating behavior” section is not part of the original C-BARQ, and therefore is not validated. However, it was deliberately added to this study to investigate this behavior in IE further. This section included some related items from C-BARQ, a question on emotional eating [25], and additional questions about voracity and food intake.

Owners were instructed to rate each item twice on a five-point Likert-type scale (0 = never/absent to 4 = always/severe), reflecting their dog’s behavior at two time points: before (past) and after (currently) seizure onset. Food intake and voracity items (from Section 7) were assessed using a different three-point scale (0 = low, 1 = normal, 2 = high).

Additionally, in 64 cases, a question regarding owners’ perception of their dogs’ quality of life (QoL) was included, rated on a five-point Likert-type scale ranging from “very poor” to “very good”.

Alongside the behavioral questionnaire, relevant clinical data were extracted from hospital records, including age at first seizure, seizure type and frequency, medication status (untreated or receiving ASMs as monotherapy or in combination), and response to treatment. Seizure types were classified as focal or generalized (including focal seizures with secondary generalization). Based on treatment response at the time of the study, dogs were classified as drug-naïve (not receiving ASMs), dogs with drug-sensitive epilepsy (DSE; receiving a single ASM and remained seizure-free or had achieved a reduction of more than 50% in seizure frequency and maintained a low seizure burden), or dogs with drug-refractory epilepsy (DRE; receiving two or more ASMs without achieving adequate seizure freedom). Overall, untreated individuals and those with DSE were collectively referred to as dogs with low seizure frequency.

### 2.3. Statistical Analysis

First, a descriptive analysis of the questionnaire data was conducted to explore the distribution of response frequencies. This analysis facilitated the grouping of responses from five-point scales into four-point scales to simplify the statistical procedures. In particular, in the scales measuring the frequency of occurrence (i.e., Sections 1, 3b, 4, 6, 7, and 8), the responses “seldom” (1 point) and “sometimes” (2 points) were combined. Similarly, in the scales assessing severity (i.e., Sections 2 and 3a), responses scoring 1 and 2 points were also grouped.

Second, comparisons of behavioral questionnaire items before and after the onset of seizures were performed using Wilcoxon’s matched-pairs signed-ranks test. Additionally, the mean score for each questionnaire subscale was also compared. For this purpose, the scores for items 1.1, 1.2, and 1.3 were reversed to align with the question formulation in the rest of the items and items 7.4 (food intake) and 7.5 (voracity) were excluded.

Third, the effects of demographical (age and sex) and clinical variables (age at first seizure, medication status, and response to treatment) during the post-seizure condition were analyzed using Mann–Whitney or Kruskal–Wallis tests. When significant effects were detected, post hoc Dunn–Bonferroni tests were conducted for multiple comparisons.

Finally, the mean values for each behavioral C-BARQ subscale were z-transformed and analyzed using principal component analysis (PCA) to reduce data complexity, followed by cluster analysis with the *k*-means algorithm. A chi-square test was used to assess the associations between cluster membership and clinical variables. The total questionnaire score (TQS), representing the sum of all scores for the post-seizures condition (excluding items 7.4 and 7.5), was compared across clusters using the Mann–Whitney test.

Statistical analyses were performed using IBM SPSS 29.0 for Windows (IBM Corp., Armonk, NY, USA), with the significance level set at 0.05. *p*-values ≤ 0.07 were interpreted as indicating a trend.

## 3. Results

### 3.1. Demographics and Clinical Data

A total of 38 males (47.3% neutered) and 32 females (68.7% spayed) were recruited. Dogs belonged to various breeds, including 37 purebred and 33 mixed-breed dogs, and aged between 1–16 years old (8.5 ± 3.7) at the time of the study. The average age at first seizure was 4.0 (±3.15) years (range 6 months to 14 years). Table 1 presents the frequency distribution of several clinical variables in the studied population. Dogs with DSE were treated with a single ASM, either phenobarbital (*n* = 17), imepitoin (*n* = 8), or levetiracetam (*n* = 4). Most dogs with DRE (89.6%) received multi-drug treatment consisting of various combinations of phenobarbital or imepitoin, primarily in combination with KBr and/or levetiracetam. In these dogs, the maximum recommended dose of phenobarbital (30–35 mg/L serum) or imepitoin (30 mg/kg every 12 h) had been reached, and combined doses of KBr or levetiracetam ranged from 10–20 mg/kg every 12 h and 20–30 mg/kg every 8 h, respectively, following consensus guidelines from the International Veterinary Epilepsy Task Force (IVETF) [27]. In five dogs, phenobarbital and imepitoin were used in combination. Overall, among the treated dogs (*n* = 58), 43 dogs (74.1%) received phenobarbital (either as monotherapy or in combination), 20 dogs (34.5%) received levetiracetam (either as monotherapy or in combination), and 17 dogs (29.3%) received imepitoin (either as monotherapy or in combination).

**Figure 1:**
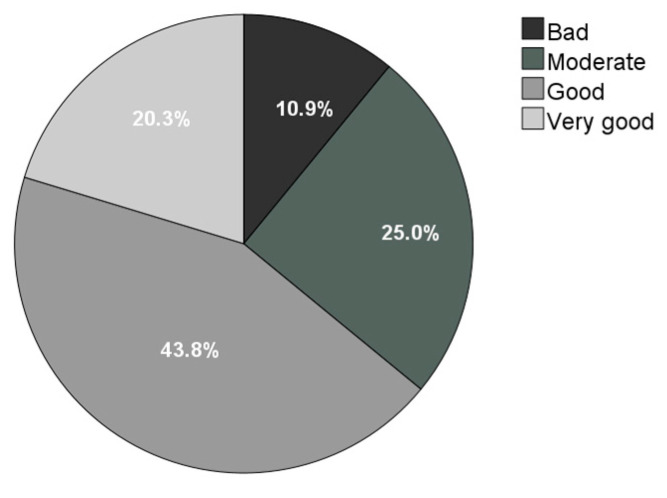
Distribution of owner-perceived quality of life (QoL) in dogs with IE (*n* = 64).

Most owners rated their dog’s QoL as “good” or “very good” (64.1%), whereas 10.9% considered QoL to be “poor” (none selected “very poor”) (Figure 1).

### 3.2. Changes in Questionnaire Scores After Seizure Onset

**Figure 2:**
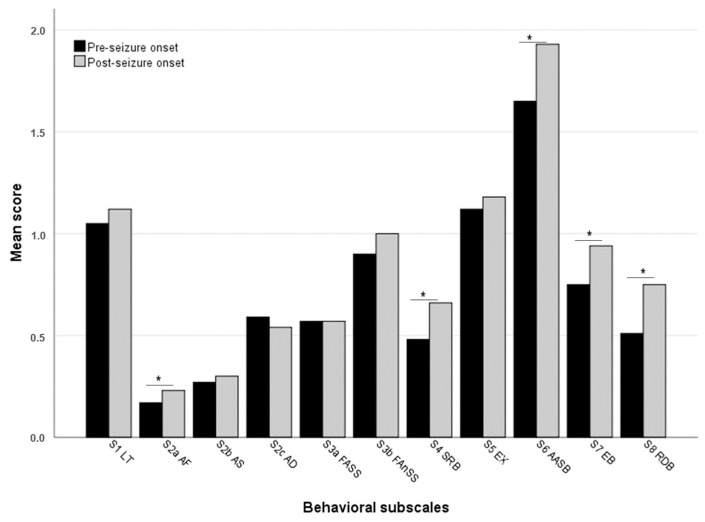
Mean questionnaire score (0–3) for each behavioral subscale before and after seizure onset. Subscales (S): LT (learning and trainability); AF (aggression toward family members); AS (aggression toward strangers); AD (aggression toward other dogs); FASS (fear/anxiety to social stimuli); FAnSS (fear/anxiety to non-social stimuli); SRB (separation-related behaviors); EX (excitability); AASB (attachment and attention-seeking behaviors); EB (eating behavior, excluding voracity and food intake items); RDB (repetitive and demented behaviors). Asterisks indicate significant differences (*p* < 0.05) between pre- and post-seizure conditions. Note: In learning and trainability, scores for items 1.1, 1.2, and 1.3 are reversed to align with the general question formulation of the rest of the subscales.

Table 2 shows the mean questionnaire scores for each item before and after seizure onset. Twelve items from different sections showed significant changes following the onset of seizures, while four additional items showed a trend (*p* ≤ 0.07). Figure 2 compares the mean questionnaire scores for each subscale or section before and after the onset of IE. Significant differences emerged for the sections on aggression towards family members, attachment and attention-seeking behaviors, separation-related behaviors, eating behavior, and repetitive and demented behaviors. In contrast, the mean scores for the sections on aggression towards strangers and dogs, fear and anxiety to social and non-social stimuli, and excitability remained unchanged after the onset of seizures, with no individual item showing any significant change. As for learning and trainability, while the mean score for the section did not change, the ability to learn new tricks easily significantly decreased after the onset of seizures.

### 3.3. Differences in Questionnaire Scores According to Demographic Variables

Table 3 shows significant differences in questionnaire scores based on the dog’s sex. Females exhibited significantly higher scores (although generally rating from “absent” to “mild”) for aggression toward other female dogs and higher scores (ranging from “mild” to “moderate”) for noise fear compared to males.

The dog’s age category was associated with differences in repetitive behaviors (*p* = 0.019), with dogs older than 9 years exhibiting these behaviors more frequently compared to those aged 5 to 9 years (*p* = 0.015).

### 3.4. Differences in Questionnaire Scores According to Seizure-Related Variables

Table 4 presents significant differences in questionnaire data according to the age at first seizure. Dogs with a late onset of seizures (>6 years) exhibited lower scores in response to the sit command and learning new tricks, as well as higher levels of fear of certain manipulations (grooming or bathing) and a higher frequency of repetitive behaviors, compared to dogs with an earlier age at first seizure (≤6 years).

Table 5 summarizes the significant differences according to seizure frequency. Seizure frequency was associated with increased fear of certain manipulations (grooming or bathing) and greater separation-related restlessness (agitation or pacing). These behaviors were more pronounced in dogs with a biweekly-to-monthly frequency (i.e., <1 month–>1 week) compared to those with semiannual-to-annual frequency (i.e., >6 months) (*p* = 0.054 and *p* = 0.009, respectively).

Table 6 presents the effect of manifesting cluster seizures or status epilepticus (SE) on neurobehavioral comorbidities. Dogs that suffered from cluster seizures or SE demonstrated a significantly reduced capacity for learning new tricks, along with a higher frequency of house-soiling and demented behaviors. Additionally, there was a trend toward increased general excitability (S5/5.4, *p* = 0.068) and pica (S7/7.2, *p* = 0.070) associated with these clinical manifestations.

### 3.5. Differences in Questionnaire Scores According to Treatment-Related Variables

Table 7 shows significant differences in questionnaire scores based on whether dogs received phenobarbital monotherapy, other ASMs monotherapies, or no medical treatment. Therefore, dogs on polytherapy (i.e., the DRE subgroup) were excluded from the analysis and only dogs with low seizure frequency were included, comprising both those with DSE and untreated individuals.

As Table 7 shows, three aggression-related items were significantly affected: dogs receiving phenobarbital showed lower scores compared to those receiving other ASMs (S2/2.2 *p* = 0.016; S2/2.4 *p* = 0.012; S2/2.5 *p* = 0.046), although the mean scores remained <1 (indicating “mild” aggression levels) in both subgroups. Drug-naïve dogs exhibited a significantly higher frequency of sitting close to or in contact with their owner compared to those treated with phenobarbital (*p* = 0.04). Additionally, dogs receiving other ASMs were less voracious than dogs in the other two groups (vs. phenobarbital group *p* = 0.019 ^a^; vs. drug-naïve group *p* = 0.024).

The number of ASMs administered (0, 1, 2 or ≥3) did not significantly affect any questionnaire item.

Table 8 presents significant differences in questionnaire items based on treatment response, comparing dogs with low seizure frequency with those diagnosed with DRE. Both habitual and separation-related house-soiling, as well as restlessness during isolation periods and pica, were significantly more frequent in dogs with DRE compared to those with low seizure frequency.

### 3.6. Principal Component Analysis and Clustering of the Behavior Questionnaire Subscales

**Figure 3:**
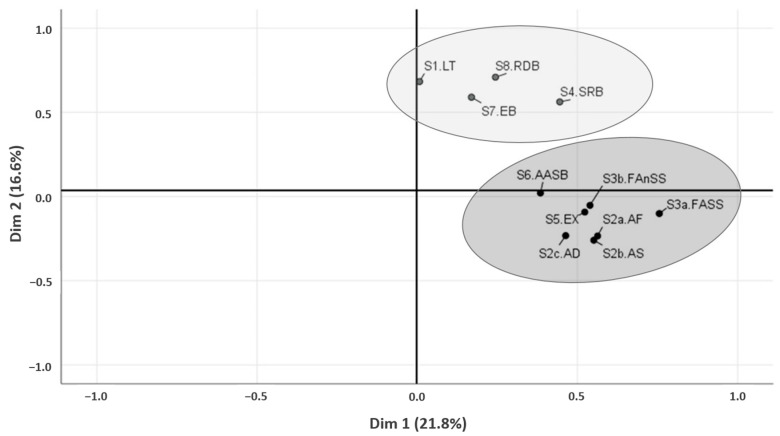
Cluster analysis of behavioral subscales. Cluster 1 (“Cognitive”, in light gray) included sections 1 (LT, learning and trainability), 4 (SRB, separation-related behaviors), 7 (EB, eating behavior) and 8 (RDB, repetitive and demented behaviors) and Cluster 2 (“Emotional”, in dark gray) comprised sections 2 (AF-AS-AD, aggression toward family members, strangers, and dogs), 3 (FASS-FAnSS, fear/anxiety to social and non-social stimuli), 5 (EX, excitability), and 6 (AASB, attachment and attention-seeking behaviors).

A total of 11 behavioral questionnaire subscales corresponding to the 8 C-BARQ sections were analyzed using PCA. The projections onto the first two dimensions accounted for 38.5% of the variance in the original data. Subsequent cluster analysis using the *k*- means algorithm revealed two distinct clusters (Figure 3). The first cluster was labeled “Cognitive” and encompassed sections 1, 4, 7, and 8 and the second cluster was labeled “Emotional” and included the remaining four sections: 2 (2a, 2b, and 2c), 3 (3a and 3b), 5, and 6. The KMO (Kaiser–Meyer–Olkin) index value was 0.53 and the Bartlett’s test of sphericity was significant (*p* < 0.001).

Chi-square tests revealed significant associations for cluster membership and two clinical variables: grouped response to treatment (*p* = 0.024) and QoL (*p* = 0.008). Belonging to Cluster 1 (“Cognitive”) was more frequently associated with DRE, as 63.2% of dogs in this cluster fell into this group. Conversely, Cluster 2 (“Emotional”) was more frequently associated with dogs exhibiting a low seizure frequency—including both those with DSE and those left untreated—comprising 66.7% of the cluster. Additionally, nearly three-quarters of dogs (73.9%) with “good” or “very good” QoL, according to their owners’ perception, belonged to Cluster 2 (“Emotional”), whereas this only occurred in 38.9% of dogs in Cluster 1 (“Cognitive”).

Finally, the Mann–Whitney test showed that the mean TQS was significantly higher (*p* < 0.001) in dogs belonging to Cluster 1 (“Cognitive”) (41.95 ± 7.7) compared to those belonging to Cluster 2 (“Emotional”) (26.4 ± 8.5).

## 4. Discussion

This cross-sectional study investigated behavioral changes following seizure onset in 70 dogs with IE using a modified owner-directed version of C-BARQ. We further investigated the impact of demographic and clinical variables on comorbidities and evaluated patient stratification based on affected behaviors. Below, we provide a comprehensive discussion that integrates these findings.

Nearly one-third of the questionnaire items (12 out of 40) exhibited statistically significant changes following seizure onset. However, the impact of seizures was not consistent across all behavioral categories. For instance, behaviors related to fear/anxiety, aggression (toward strangers and other dogs), and excitability showed no changes following the onset of epilepsy. This contrasts with previous cross-sectional studies that reported increases in both social and non-social anxiety- and fear-related behaviors [12,14,26] as well as in dog-directed aggression [14], when comparing pre- and post-seizure conditions.

The absence of a significant effect on these behavioral categories may be attributed to the considerable heterogeneity within our study population regarding anxiety/fear and aggression, as reflected by the large standard deviations in the corresponding questionnaire items. Only aggression towards family members when approaching the dog while it was resting was significantly affected after the onset of seizures. Nonetheless, mean scores for social fear and anxiety, as well as for all types of aggression, remained relatively low both before and after the onset of epilepsy. Despite this, both family- and stranger-directed aggression decreased in dogs treated with phenobarbital compared to those receiving other ASMs, possibly due to its anxiolytic effects [29]. Supporting this observation, one study reported improvements in stranger-directed and non-social fear after 90 days of phenobarbital treatment [7].

Additionally, factors beyond disease-related mechanisms could influence these emotional responses, such as sex. In our study, female dogs more frequently exhibited aggression toward other females when approached, aligning with previous evidence indicating that intrasexual aggression (i.e., male–male or female–female aggression) is commonly observed across species in nature [30], including in dogs [31]. Furthermore, female dogs also displayed a higher frequency of fear responses to sudden or loud noises compared to males. A sex-related predisposition to a higher prevalence of phobias—including noise phobia—in female dogs has been previously documented in a large retrospective case series [32].

Consistent with our findings, excitability—as measured by the C-BARQ—did not increase following seizure onset relative to the pre-seizure condition, as reported by Watson and colleagues [14]. However, higher levels of excitability and chasing behavior (the latter not analyzed in our study) have been reported in dogs with IE, and these behaviors were reduced following a ketogenic diet [16]. In our study, excitability scores were relatively high compared to other behavioral categories. However, to determine whether epileptic dogs truly exhibit heightened excitability—even before seizure onset—it would be necessary to compare these scores with those from a control group. In fact, the aforementioned study conducted such a comparison and found that the only significant difference was in trainability, which was lower in dogs with IE than in healthy dogs [14].

On the other hand, we found that the category of attachment and attention-seeking behaviors was notably affected. This finding aligns with previous evidence reporting an increase in these behaviors following the onset of IE in dogs [14]. Additionally, separation-related behaviors—closely associated with both the attachment and the anxiety/fear emotional systems—were also affected in our study as well as in others [12,13,26].

The tendency toward clinginess may be interpreted as a care-soliciting (etepimeletic) behavior during the prodromal phase, in response to acute sensations preceding a seizure [33], as well as during the interictal period, where it may reflect comorbid anxiety. Notably, untreated dogs exhibited a greater tendency to remain close to or in direct contact with their owner compared to those receiving phenobarbital treatment. This finding supports the anxiolytic effects of phenobarbital, as previously mentioned [7,14], and underscores the importance of monitoring attachment behaviors in untreated dogs as potential indicators of heightened anxiety.

The category of eating behavior was also notably affected following seizures, including emotional eating, which did not appear to be influenced by clinical variables such as medication status or treatment response. In line with this, the odds of developing eating disorders are higher in people with epilepsy compared to those without epilepsy [2]. However, voracity was lower in treatment-responsive dogs receiving ASMs other than phenobarbital (i.e., imepitoin or levetiracetam) compared to those treated solely with phenobarbital or left untreated. Polyphagia is a well-documented adverse effect of first-line ASMs; however, a comparative study in epileptic dogs found that phenobarbital induced an increase in appetite more frequently than imepitoin [34]. Conversely, appetite loss has been reported with levetiracetam [24].

Finally, although not all items within the rest of the categories were significantly affected following seizures, a decline in trainability—manifested as difficulty in learning new tricks—was observed, along with an increase in dementia-like behaviors such as staring into space, aimless wandering, and disorientation. Similarly, previous studies on canine IE have observed decreased trainability [12,14,17] and increased signs of abnormal perception (ref. [12], equivalent to our demented behaviors) after epilepsy onset.

Both learning and memory deficits—which can also affect house-soiling—as well as disorientation and apparent confusion, are typical signs of cognitive impairment in elderly dogs. However, cognitive impairment in dogs with IE appears to differ from age-related cognitive dysfunction syndrome (CDS) in several ways. For instance, in addition to those impairments, reduced social interaction and altered sleep patterns are hallmark signs of CDS [35], but not commonly seen in epileptic dogs.

Beyond differences in clinical presentation, the largest epidemiological study to date on canine cognitive dysfunction found that memory and orientation deficits in IE appeared at a younger age (under 4 years), whereas in non-epileptic dogs (i.e., controls), the risk typically increased after 10 years of age. Moreover, no significant progression of cognitive dysfunction was observed over time in epileptic dogs compared to controls, suggesting that these deficits may be present from the onset of seizures. This supports the idea that epilepsy may interfere with the normal development of brain networks responsible for cognition [19] (see below).

Despite the relatively static nature of cognitive dysfunction in canine IE, it is possible that aging may exacerbate pre-existing impairments. In this regard, we observed that dogs with a late onset of seizures (after 6 years of age) exhibited lower trainability, particularly in their response to the “sit” command and their ability to learn new tricks. Moreover, both late seizure onset and being older than 9 years at the time of the study—potentially related variables—were associated with an increase in repetitive behaviors. While mild perseveration can be a normal aspect of aging, patients with Alzheimer’s disease show high rates of repetitive behaviors [36]. Similarly, repetitive and aimless behaviors have been observed in aged dogs with CDS [37]. These findings underscore the need for further research investigating the effects of aging in dogs with IE, particularly in comparison to age-matched controls.

Principal component analysis (PCA) followed by clustering of the behavioral questionnaire subscales resulted in the identification of two distinct clusters, labeled “Cognitive” and “Emotional”, referring to the nature of the behavioral categories included in each, more closely related to cognitive impairment or emotional disorders, respectively. These findings suggest that neurobehavioral comorbidities in canine IE may be broadly grouped into two profiles, depending on whether the affected domains are primarily cognitive- or emotion-related. Moreover, these clusters appeared to be associated with treatment response, highlighting the clinical relevance of the identified neurobehavioral profiles. Dogs in the Cognitive cluster were more commonly affected by drug-resistant epilepsy, while those in the Emotional cluster were primarily dogs with low seizure frequency, including both drug-sensitive epilepsy and untreated cases.

In particular, the Cognitive cluster included behavioral categories related to learning and trainability as well as to repetitive and demented behaviors—core signs of cognitive dysfunction in IE, as described above. It also encompassed the section on separation-related behaviors. While vocalizations during isolation periods are frequently associated with the activation of anxiety/fear and/or attachment systems, as previously noted, other signs such as restlessness (agitation or pacing) and house-soiling when left alone may not be purely emotional in origin. Instead, they could also reflect underlying cognitive impairment, as has been described in CDS in elderly dogs [35]. Interestingly, both restlessness and house-soiling when left alone were higher in dogs with refractory epilepsy. The Cognitive cluster also included the section on eating behavior, referring specifically to the compulsive nature of certain behaviors, particularly pica, which was likewise more common in dogs with refractory epilepsy.

Although restlessness, house-soiling, and pica may all represent adverse effects of ASMs—particularly in dogs receiving polytherapy, as is typical in drug-resistant cases—the number of ASMs received (ranging from one to three or more) did not significantly affect these items, nor any others in the questionnaire. This contrasts with a previous study, which reported that treatment with two to three ASMs, and specific medications such as zonisamide (not used in the present study) and potassium bromide, were associated with reduced training performance [17], suggesting that these effects could, at least in part, be attributed to cognitive impairment. The absence of an association between the number of ASMs and the frequency or severity of problematic behaviors in our study argues against a purely pharmacological explanation.

A plausible alternative is that chronic and poorly controlled epileptic activity may exert a more profound impact on brain networks involved in cognition, particularly those related to learning, memory, and spatial orientation [38,39]. Indeed, in our study, the occurrence of cluster seizures or status epilepticus (SE) was associated with difficulties in learning new tricks and house-soiling, as well as with showing demented behaviors. In people with epilepsy, both cluster seizures and, especially, SE have long been recognized as major risk factors for cognitive impairment [40]. Among the brain regions most susceptible to damage induced by SE are the cerebral cortex, the claustrum, and, in particular, the hippocampus [41,42].

In contrast, the Emotional cluster grouped behavioral categories more clearly aligned with discrete emotional systems—such as fear, frustration, and attachment [43]—including fear/anxiety-related signs, aggression, attention or contact-seeking behavior, and excitability. This emotional profile may represent a different neurobiological substrate, potentially involving the hyperexcitability of limbic or hypothalamic structures or the dysregulation of neurotransmitter systems (e.g., serotonin and noradrenaline) known to modulate emotional reactivity [44,45]. A previous study using the C-BARQ found that seizure frequency and severity were not correlated with anxiety-related behavior in dogs with IE; however, dogs receiving polytherapy exhibited increased fear and anxiety when being groomed [13]. Similarly, in our study, a higher seizure frequency was also associated with increased fear of bathing or grooming by the owner.

Therefore, the presence of both profiles in dogs with IE, and their apparent association with treatment response, suggest individual variability in the expression and neurobiological underpinnings of comorbid behaviors. The significant association between cluster membership and owner-reported QoL further highlights the clinical relevance of these neurobehavioral patterns. Owners of dogs within the Emotional cluster were more likely to rate their dogs’ QoL as “good” or “very good,” whereas such ratings were markedly less frequent among dogs in the Cognitive cluster. This may reflect the more pervasive and functionally limiting nature of cognitive-related behavioral issues—such as dementia-like behaviors, restlessness, or house-soiling—which may interfere more severely with the dog’s autonomy, daily functioning, and the quality of owner–dog interactions.

Our findings on poorer QoL associated with the Cognitive cluster are not surprising, considering that more severe and uncontrolled seizures—characteristic of refractory cases—lead to significantly lower QoL in both humans [46,47] and dogs [48,49,50] compared to less severe or more manageable forms of epilepsy. Interestingly, a prospective study in children with new-onset epilepsy showed that specific subgroups with lower health-related QoL exhibited disproportionately greater impairments in cognitive functioning compared to emotional or physical domains [51].

Finally, the significantly higher total questionnaire scores observed in dogs from the Cognitive cluster support the interpretation that this profile is not only qualitatively, but also quantitatively more severe. Taken together, these findings suggest that the Cognitive profile is associated with greater neurobehavioral burden, poorer seizure control, and a lower perceived QoL. Nevertheless, anxiety-related behaviors—broadly defined—should also be carefully monitored in epileptic dogs exhibiting an Emotional profile. Furthermore, some individuals may present with concurrent impairments in both the cognitive and emotional domains.

Thus, future research on neurobehavioral comorbidities in canine IE should aim to identify discrete phenotypes and their associated clinical correlates, in order to detect vulnerable individuals—an approach that is already being pursued in human epilepsy research [1]. Once identified, these dogs may benefit from targeted behavioral, cognitive, and pharmacological interventions [4,14,52], as well as from dietary [16] and potential microbiota-based interventions such as fecal transplantation [53], in addition to seizure management strategies. This multimodal approach to treating canine epilepsy and its comorbidities underscores the need for multidisciplinary collaboration, involving neurologists and general practitioners alongside specialists in behavior medicine, as previously emphasized by others [52].

A limitation of this study is that the characterization of neurobehavioral comorbidities was based solely on data obtained from the C-BARQ questionnaire. While this is a validated and standardized tool for investigating problem behaviors in dogs, a more comprehensive behavioral assessment—including a detailed history and the use of additional validated instruments such as the canine ADHD questionnaire [54], the CCDR (Canine Cognitive Dysfunction Rating) [55], or the CADES (CAnine DEmentia Scale) [56]—as well as cognitive tests (e.g., memory test [18] or spatial discrimination test [57]), would be necessary to more accurately characterize individual patients. In addition to the inherent limitations of owner-reported data, the fact that owners had to score questionnaire items before and after seizure onset could had led to confusion, particularly in cases where there was a prolonged interval between seizure onset and survey participation. Considering this, further longitudinal studies are recommended to monitor behavioral changes from the onset of seizures in order to better understand the development and progression of these comorbidities in canine IE.

The use of univariate statistical analyses also represents a limitation in the first part of the study, as these methods assess only one variable at a time (e.g., age, cluster seizures, or treatment response), offering a limited view of complex phenomena such as epilepsy and its comorbidities. A larger sample size in future studies may facilitate the application of multivariate approaches (e.g., MANOVA), enabling the simultaneous examination of multiple variables and their interrelationships. Nevertheless, the use of PCA and subsequent cluster analysis in the second part of the study complemented the initial univariate analyses, allowing for the classification of individuals based on patterns observed across behavioral data. Although the KMO value was at the lower acceptable threshold (0.53), Bartlett’s test was significant and the resulting factor structure was clinically interpretable.

## 5. Conclusions

This study provides new insights into the complexity of neurobehavioral comorbidities in dogs with IE, highlighting both categorical and dimensional changes following seizure onset. Behavioral changes were not uniformly distributed across all domains: while fear/anxiety, aggression, and excitability remained relatively stable, significant alterations were observed in attachment- and separation-related behaviors, eating behavior (including emotional eating), and signs indicative of cognitive impairment—such as reduced trainability or dementia-like behaviors. Notably, these changes were not solely attributable to ASMs, suggesting the involvement of epilepsy-related processes, including refractoriness and seizure-related brain damage, such as that caused by cluster seizures and status epilepticus.

Moreover, our findings offer preliminary evidence supporting the existence of two distinct neurobehavioral profiles—Cognitive and Emotional—in canine IE. These profiles were not only behaviorally distinct but also clinically relevant: the Cognitive cluster was associated with a higher total questionnaire score, poorer seizure control (drug-resistant epilepsy), and lower owner-perceived QoL, while the Emotional cluster was more frequently observed in dogs with low seizure frequency and was associated with higher QoL scores. These findings underscore the value of stratifying patients based on comorbid cognitive and emotional domains, paving the way for more individualized and targeted clinical management in canine epilepsy.

## Figures and Tables

**Table 1:** Distribution of clinical variables in the dogs with IE (*n* = 70).

Variable	Categories	*n* (%)
Age at first seizure	<6 months	1 (1.4%)
	6 months–6 years	51 (72.9%)
	>6 years	18 (25.7%)
Interictal period length	>6 months	27 (38.6%)
	<6 months–>1 month	18 (25.7%)
	<1 month–>1 week	22 (31.4%)
	<1 week	3 (4.3%)
Seizure type	Generalized (primary or secondary)	66 (94.3%)
	Focal	4 (5.7%)
Cluster/status epilepticus	Yes	46 (65.7%)
	No	24 (34.3%)
Response to treatment	Drug-naïve (untreated epilepsy)	12 (17.1%)
	Drug-sensitive epilepsy (DSE)	29 (41.4%)
	Drug-refractory epilepsy (DRE)	29 (37.5%)

**Table 2:** Mean (±SD) scores (0–3) for questionnaire items before and after seizure onset (*n* = 70).

Section	Item (Abbreviated Question)	Mean (±SD) Before	Mean (±SD) After	*p*-Value ^1^
1. Learning and trainability	1.1. When off leash, returns immediately when called	1.8 (±1.0)	1.9 (±0.9)	NS
	1.2. Obeys the “sit” command immediately	1.9 (±1.2)	1.9 (±1.1)	NS
	1.3. Learns new tricks easily	1.8 (±1.0)	1.6 (±1.0)	0.053
	1.4. Eliminate in inappropriate areas (not alone at home)	0.6 (±0.9)	0.8 (±0.9)	0.065 (trend)
2. Aggression	2a. Aggression toward family members			
	2.1. When verbally corrected/punished	0.2 (±0.6)	0.2 (±0.6)	NS
	2.2. When toys, bones or others are taken away	0.3 (±0.5)	0.3 (±0.5)	NS
	2.3. When approached directly while eating	0.1 (±0.5)	0.2 (±0.6)	NS
	2.4. When approached directly while resting	0.07 (±0.3)	0.1 (±0.4)	0.025
	2b. Aggression toward strangers			
	2.5. When approached directly by an adult on leash	0.2 (±0.5)	0.3 (±0.6)	NS
	2.6. When approached directly by a child on leash	0.2 (±0.6)	0.2 (±0.6)	NS
	2.7. When unfamiliar people try to touch/pet	0.3 (±0.6)	0.3 (±0.7)	NS
	2.8. Toward unfamiliar people visiting home	0.3 (±0.6)	0.4 (±0.6)	NS
	2c. Aggression toward other dogs			
	2.9. When approached by unfamiliar male dog on leash	0.7 (±0.9)	0.6 (±0.85)	NS
	2.10. When approached by unfamiliar female dog on leash	0.5 (±0.8)	0.4 (±0.7)	NS
3. Fear and anxiety	3a. Fear and anxiety to social stimuli			
	3.1. When approached by unfamiliar adult	0.6 (±0.9)	0.5 (±0.8)	NS
	3.2. When approached by unfamiliar child	0.5 (±0.8)	0.4 (±0.75)	NS
	3.3. When unfamiliar people try to touch/pet	0.5 (±0.9)	0.5 (±0.9)	NS
	3.4. When approached by unfamiliar dog	0.6 (±09)	0.6 (±0.8)	NS
	3.5. When groomed/bathed by household member	0.7 (±0.9)	0.8 (±0.85)	NS
	3b. Fear and anxiety to non-social stimuli			
	3.6. In response to sudden/loud noises	1.0 (±1.0)	1.1 (±1.0)	NS
	3.7. In response to strange/unfamiliar objects	0.6 (±0.8)	0.6 (±0.8)	NS
	3.8. During thunderstorms, fireworks or similar	1.1 (±1.2)	1.2 (±1.2)	NS
4. Separation-related behaviors	4.1. Restlessness, agitation, pacing	0.6 (±0.8)	0.9 (±1.0)	0.020
	4.2. Whining, barking and/or howling	0.6 (±0.8)	0.8 (±1.0)	0.022
	4.3. Chewing/scratching at house elements.	0.3 (±0.6)	0.4 (±0.8)	NS
	4.4. House-soiling	0.3 (±0.7)	0.4 (±0.7)	0.072 (trend)
5. Excitability	5.1. When playing with household members	1.2 (±0.9)	1.2 (±0.8)	NS
	5.2. When doorbell rings	1.4 (±1.1)	1.6 (±1.1)	NS
	5.3. Just before being taken for a walk	1.4 (±1.0)	1.5 (±1.0)	NS
	5.4. General excitation (no specific context)	0.3 (±0.6)	0.4 (±0.6)	NS
6. Attachment and attention-seeking behaviors	6.1. Tends to follow owners at home	1.7 (±0.9)	2.1 (±0.9)	0.008
	6.2. Tends to sit close to/in contact with owners	1.8 (±0.9)	2.0 (±0.85)	0.005
	6.3. Tends to nudge, nuzzle or paw for attention	1.5 (±1.0)	1.7 (±1.0)	0.027
7. Eating behavior	7.1. Steals food	0.9 (±0.9)	1.0 (±1.0)	0.017
	7.2. Pica	0.6 (±0.7)	0.75 (±0.95)	0.070 (trend)
	7.3. Emotional eating	0.8 (±0.8)	1.1 (±1.0)	0.007
	7.4. Amount of food intake *	0.9 (±0.5)	1.2 (±0.6)	0.012
	7.5. Voracity *	1.1 (±0.7)	1.3 (±0.8)	0.007
8. Repetitive and demented behaviors	8.1. Repetitive behaviors (fly snapping, shadow chasing, circling, tail chasing…)	0.6 (±0.8)	0.8 (±1.0)	0.066 (trend)
	8.2. Demented behaviors (staring into space, aimless wandering, disorientation…)	0.4 (±0.75)	0.7 (±1.0)	0.002

^1^ Wilcoxon test. * Note: these items were scored from 0 to 2. NS: non-significant.

**Table 3:** Effect of sex on questionnaire scores (0–3) in dogs with IE.

Section/Item (Abbreviated)	Sex	Mean (±SD)	*p* Value ^1^
S2/2.10. Female dog aggression	Male	0.2 (±0.5)	0.001
	Female	0.7 (±0.8)	
S3/3.6. Noise fear/anxiety	Male	0.9 (±0.9)	0.041
	Female	1.4 (±1.1)	

^1^ Mann–Witney test. Only significant differences (*p* < 0.05) are shown.

**Table 4:** Effect of the age at first seizure on questionnaire scores (0–3) in dogs with IE.

Section/Item (Abbreviated)	Age at First Seizure	Mean (±SD)	*p* Value ^1^
S1/1.2. Obeying to “sit”	≤6 years	2.1 (±1.1)	
	>6 years	1.4 (±1.1)	0.027
S1/1.3. Learning new tricks	≤6 years	1.75 (±1.0)	
	>6 years	1.0 (±0.9)	0.005
S3/3.5. Fear of grooming/bathing	≤6 years	0.7 (±0.9)	
	>6 years	1.1 (±0.6)	0.018
S8/8.1. Repetitive behaviors	≤6 years	0.6 (±0.8)	
	>6 years	1.3 (±1.0)	0.005

^1^ Mann–Whitney test. Only significant differences (*p* < 0.05) are shown.

**Table 5:** Effect of the seizure frequency on questionnaire scores (0–3) in dogs with IE.

Section/Item (Abbreviated)	Seizure Frequency	Mean (±SD)	*p* Value ^1^
S3/3.5. Fear of grooming/bathing	>6 months <6 months–>1 month	0.6 (±0.7) ^a^ 0.6 (±0.8)	
	<1 month–>1 week <1 week	1.2 (±1.0) ^b^ 0.7 (±0.6)	0.034
S4/4.1. Restlessness (separation-related)	>6 months <6 months–>1 month	0.5 (±0.8) ^a^ 0.9 (±0.9)	
	<1 month–>1 week <1 week	1.4 (±1.0) ^b^ 1.0 (±1.0)	0.017

^1^ Kruskal–Wallis test. Only significant differences (*p* < 0.05) are shown. Different letters (a, b) mean significant differences between subgroups.

**Table 6:** Effect of suffering cluster seizures or status epilepticus (SE) on questionnaire scores (0–3) in dogs with IE.

Section/Item (Abbreviated)	Cluster or SE	Mean (±SD)	*p* Value ^1^
S1/1.3. Learning new tricks	Yes	1.4 (±1.0)	
	No	1.9 (±1.0)	0.027
S1/1.4. House-soiling	Yes	1.0 (±1.0)	
	No	0.3 (±0.6)	0.003
S8/8.2. Demented behaviors	Yes	0.9 (±1.1)	
	No	0.3 (±0.7)	0.009

^1^ Mann–Whitney test. Only significant differences (*p* < 0.05) are shown.

**Table 7:** Effect of medication status on questionnaire scores (0–3) in dogs with low seizure frequency (*n* = 41).

Section/Item (Abbreviated)	Medication Status	Mean (±SD)	*p* Value ^1^
S2/2.2. Resource-related aggression	Drug-naïve	0.4 (±0.7)	0.015
	Phenobarbital	0.1 (±0.35) ^a^	
	Other ASMs	0.6 (±0.6) ^b^	
S2/2.4. Resting-related aggression	Drug-naïve	0.1 (±0.3)	0.015
	Phenobarbital	0.05 (±0.2) ^a^	
	Other ASMs	0.4 (±0.7) ^b^	
S2/2.5. Adult-directed aggression	Drug-naïve	0.2 (±0.6)	0.033
	Phenobarbital	0.2 (±0.5) ^a^	
	Other ASMs	0.5 (±0.6) ^b^	
S6/6.2. Sit in contact with owner	Drug-naïve	2.6 (±0.7) ^a^	0.044
	Phenobarbital	1.9 (±0.8) ^b^	
	Other ASMs	2.1 (±1.0)	
S7/7.5. Voracity *	Drug-naïve	1.6 (±0.8) ^a^	0.009
	Phenobarbital	1.5 (±0.7) ^a^	
	Other ASMs	0.8 (±0.8) ^b^	

^1^ Kruskal–Wallis test. Only significant differences (*p* < 0.05) are shown. Different letters (a, b) mean significant differences between subgroups. * Note: this item was scored from 0 to 2.

**Table 8:** Effect of treatment response on questionnaire scores (0–3) in dogs with IE.

Section/Item (Abbreviated)	Treatment Response	Mean (±SD)	*p* Value ^1^
S1/1.4. House-soiling	DRE	1.0 (±1.0)	
	non-DRE	0.6 (±0.8)	0.045
S4/4.1. Restlessness (separation-related)	DRE	1.3 (±1.0)	
	non-DRE	0.6 (±0.9)	0.001
S4/4.4. House-soiling (separation-related)	DRE	0.7 (±0.9)	
	non-DRE	0.3 (±0.5)	0.052
S7/7.2. Pica	DRE	1.1 (±1.1)	
	non-DRE	0.5 (±0.8)	0.010

^1^ Mann–Whitney test. Only significant differences (*p* < 0.05) are shown. DRE: drug-resistant epilepsy group; non-DRE: untreated and drug-sensitive epilepsy groups.

## Data Availability

The original contributions presented in this study are included in the article. Further inquiries can be directed to the corresponding author.

## Supplementary Materials

The following supporting information can be downloaded at: https://www.mdpi.com/article/10.3390/ani15111592/s1, Informed Consent File.
