# Supplementary material for: Neurobehavioral Comorbidities in Canine Idiopathic Epilepsy: New Insights into Cognitive and Emotional Domains
Source: Animals (Basel). 2025 May 29;15(11):1592. doi: 10.3390/ani15111592 (PMC12153923; doi:10.3390/ani15111592)
Supplement: Supplementary file 1 [file animals-15-01592-s001.zip › animals-3649324-supplementary.pdf]

Hospital
Veterinario
Universidad Zaragoza

CONSENTIMIENTO INFORMADO

Participación en el Estudio ALTERACIONES COGNITIVO CONDUCTUALES Y ESTRATEGIAS ÓMICAS EN EL DIAGNÓSTICO Y TRATAMIENTO DE LA EPILEPSIA IDIOPÁTICA CANINA

Yo, ____ con DNI ________________________, como propietario/a del perro/a ___________________, de raza _________________________, con fecha de nacimiento_____________, estado reproductivo ___________________________ y número de microchip_________________________, declaro que he recibido y entendido la información proporcionada sobre el procedimiento experimental y sobre los riesgos que conlleva, y los acepto.

Asimismo, declaro que he leído este formulario de consentimiento, que he tenido la oportunidad de discutir este estudio con el investigador que me lo ha presentado y que mis preguntas han sido contestadas en un lenguaje que entiendo. Los riesgos y los beneficios han sido explicados. Pienso que no he sido indebidamente influenciado por ningún miembro del equipo de estudio para participar y entiendo que se me dará una copia de este formulario de consentimiento después de firmarlo. Entiendo que mi participación en este estudio es voluntaria y que puedo optar por retirarme en cualquier momento. Estoy libremente de acuerdo en participar en este estudio de investigación y entiendo que la información de mis datos personales se mantendrá confidencial. Estoy de acuerdo en ser contactado con relación a este estudio.

Por todos estos motivos, autorizo al Servicio de Neurología del Hospital Veterinario de la Universidad de Zaragoza a llevar a cabo el procedimiento experimental que previamente se me ha explicado, necesario para ALTERACIONES COGNITIVO CONDUCTUALES Y ESTRATEGIAS ÓMICAS EN EL DIAGNÓSTICO Y TRATAMIENTO DE LA EPILEPSIA IDIOPÁTICA CANINA, para lo cual se recogerán muestras de heces y sangre antes y después de 4 semanas de haber iniciado el tratamiento indicado clínicamente para la epilepsia.

Asimismo, autorizo la utilización de fotografías, vídeos o datos analíticos de mi perro para su difusión con fines exclusivamente científicos o divulgativos.

En Zaragoza, a ____ de ________________ de ______.

Nombre y apellidos:
DNI:

El abajo firmante declara que ha explicado los detalles relevantes de este estudio para el participante nombrado anteriormente y cree que el participante ha entendido y ha dado su consentimiento de una manera libre.

Nombre y apellidos:

DNI:
